# Attention capture by own name decreases with speech compression

**DOI:** 10.1186/s41235-024-00555-9

**Published:** 2024-05-12

**Authors:** Simon Y. W. Li, Alan L. F. Lee, Jenny W. S. Chiu, Robert G. Loeb, Penelope M. Sanderson

**Affiliations:** 1 School of Psychological Science, The University of Western Australia, Perth, Australia; 2 Department of Psychology, Lingnan University, Hong Kong SAR, China; 3 School of Psychology, The University of Queensland, Brisbane, Australia; 4 Department of Anesthesiology, University of Florida School of Medicine, Gainesville, USA; 5 School of Information Technology and Electrical Engineering, The University of Queensland, Brisbane, Australia; 6 School of Clinical Medicine, The University of Queensland, Brisbane, Australia

**Keywords:** Attention capture, Time-compressed speech, Cocktail party effect

## Abstract

Auditory stimuli that are relevant to a listener have the potential to capture focal attention even when unattended, the listener’s own name being a particularly effective stimulus. We report two experiments to test the attention-capturing potential of the listener’s own name in normal speech and time-compressed speech. In Experiment 1, 39 participants were tested with a visual word categorization task with uncompressed spoken names as background auditory distractors. Participants’ word categorization performance was slower when hearing their own name rather than other names, and in a final test, they were faster at detecting their own name than other names. Experiment 2 used the same task paradigm, but the auditory distractors were time-compressed names. Three compression levels were tested with 25 participants in each condition. Participants’ word categorization performance was again slower when hearing their own name than when hearing other names; the slowing was strongest with slight compression and weakest with intense compression. Personally relevant time-compressed speech has the potential to capture attention, but the degree of capture depends on the level of compression. Attention capture by time-compressed speech has practical significance and provides partial evidence for the duplex-mechanism account of auditory distraction.

## Significance Statement

Traditional auditory alerts such as medical alarms rely on abstract sounds to attract listeners’ attention. Although they are effective as urgent warnings, such alerts can disrupt listeners’ focus of attention, especially when occurring frequently, and can impair general task performance. Appropriately designed auditory alerts should attract listeners’ attention only when necessary. One way to achieve this is to exploit unconscious-level processing to help listeners decide whether or not to shift their focal attention. Our prior work on time-compressed speech has shown that people can accurately identify the content of time-compressed speech phrases. However, it is unknown how effectively time-compressed speech phrases might draw listeners’ attention in an unconscious manner. Given that hearing one’s own name has a strong tendency to attract focus of attention, we have designed an experimental paradigm to investigate whether one’s attention on an ongoing task would be diverted by hearing one’s own name in time-compressed formats. The current study validates a novel task paradigm for measuring attention capture by own name, then applies this method to test for attention capture at various speech compression rates. This is an important first step towards establishing whether professionally relevant time-compressed speech would be effective in capturing a professional’s attention—for example, to patient deterioration in healthcare settings. This research is particularly timely as speech-based alerts have recently been advocated by healthcare human factors researchers.

## Introduction

Attention capture by unattended auditory stimuli has a long history of investigation (Cherry, 1953; Moray, 1959) and replication (Röer & Cowan, 2021; Wood & Cowan, 1995). It continues to stimulate both theoretical development (Hughes, 2014) and empirical investigation (Conway et al., 2001; Naveh-Benjamin et al., 2014; Röer et al., 2013; Shapiro et al., 1997; Yang et al., 2013). In this paper, we explore whether unattended *time-compressed* speech with personal relevance has the same attention-capturing properties as uncompressed speech. Our research is motivated by the question of whether unattended time-compressed speech alerts, when heard by domain experts in professional contexts, will capture attention as effectively as uncompressed speech alerts can. The present study is an initial investigation of the principle, before moving to a professional context such as health care.

Involuntary auditory distraction can come from unattended stimuli that are abstract (e.g. a tone) or semantically meaningful (e.g. words). Jones & Macken (1993) showed that changes in discrete abstract tones can distract attention from a visual memory task, resulting in impaired recall. In a dichotic listening task, Cherry (1953) showed that listeners noticed changes in the physical features of unattended speech messages, but not changes in message content. However, Moray (1959) showed that 33% of participants shadowing a message in one ear responded to a command in the unattended ear that was preceded by their own name. Replications show that around 30 to 40% of participants performing shadowing tasks report hearing their own name in the unattended ear (Röer & Cowan, 2021; Wood & Cowan, 1995).

Two psychological processes have been proposed to explain distraction by unattended auditory stimuli: interference-by-process and attentional capture. Hughes (2014) proposes that these two processes are not mutually exclusive and names them the *duplex mechanism*. *Interference-by-process* indicates that irrelevant sounds with constantly changing acoustic properties will produce involuntary processing that interferes with the processing required for a focal task such as serial recall (Hughes et al., 2005). *Attentional capture* indicates that an auditory stimulus can shift attention away from another task. Attentional capture can be specific or aspecific. *Specific* attentional capture accounts for distraction from stimuli that have specific relevance to the listener, such as one’s name, which is also referred to as the cocktail party effect (CPE). *Aspecific* attentional capture accounts for distraction coming from deviations in sound quality alone.

Recent evidence suggests that specific stimuli such as one’s own name cause more auditory distraction than aspecific stimuli that simply deviate from context. Röer and Cowan (2021) showed that own name captured attention, resulting in errors in a shadowing task, whereas unexpected words deviating from their semantic context were rarely detected and did not affect shadowing performance. Furthermore, Röer et al. (2013) showed that serial memory performance was worse in the presence of own name than in the presence of other names. They also found habituation, where participants made progressively fewer serial memory errors when they repeatedly heard their own name.

Applications of auditory distraction research have mostly focused on ways to reduce the disruptive effects of auditory stimuli on primary task performance (Dalton & Hughes, 2014; Hughes, 2014). In contrast, our motivation is to exploit attentional capture for practical situations in which shifts of attention are required. For example, medical alarms are widely used in hospitals to alert clinicians of potential problems with patients or equipment. However, healthcare workers can become desensitized to medical alarms, resulting in practices that could compromise patient safety (Ruskin & Hueske-Kraus, 2015). Woods (1995) suggests that an alternative to using alarms is to create sounds that can attract preattentive- or unconscious-level processing to help listeners decide whether or not to shift their focal attention. The function of preattentive processing is not to draw the listener’s focal attention unnecessarily, but only when the changes are professionally important to the listener.

The use of speech in auditory alerts, rather than the abstract sounds typical of current medical alarms, has been recently advocated by healthcare human factors researcher (Roche et al., 2021; Sanderson et al., 2019). We have shown that people can quickly learn and accurately identify the content of time-compressed speech phrases reflecting patient vital signs in different languages such as Cantonese (Li et al., 2017) and English (Sanderson et al., 2019). Srbinovska et al. (2021) have shown that time-compressed speech is easily understood by trained listeners, but not untrained listeners; this is desirable in clinical situations where the speech content is meant to be understood by the trained (e.g. clinicians), but not the untrained (e.g. relatives of patients), in order to avoid unnecessary stress or anxiety. Time-compressed speech has also been found to better than medical alarms in supporting multiple-patient monitoring (Deschamps et al., 2022).

However, it is unknown how effectively uncompressed speech phrases indicating patient deterioration might draw attention from a visual ongoing task, in the preattentive manner recommended by Woods (1995) and whether time-compressed speech alerts would be as effective as uncompressed speech at doing so. Our ultimate goal is to determine whether time-compressed speech that is professionally relevant would be effective in capturing a professional’s attention.

In the studies to be reported, we investigate whether the sound of the participant’s own name will degrade performance on a concurrent visual forced choice reaction time task (Experiment 1). The choice of tasks was aimed at creating an experimental paradigm that involved cross-modal sharing of attention. In the context of healthcare work, clinicians often have to perform cross-modal attention-sharing tasks. For example, monitoring patient’s health status via sound alerts (auditory) while reading patient’s record (visual). However, the current study is not intended to be representative of clinical tasks per se, but serves as a novel task paradigm for measuring attention capture by own name, and then applies this method to test for attention capture.

Previous research reviewed above, using different paradigms and different measures of distraction, suggests that unattended but personally relevant auditory stimuli should capture attention from a primary visual task. Therefore, we also investigate whether a *time-compressed* version of the participant’s own name still captures attention from the visual task (Experiment 2). Positive results would support the specific attentional capture mechanism of the duplex-mechanism account and would have practical significance by showing that personally relevant time-compressed speech has the same attention-capturing properties as personally relevant uncompressed speech.

## Experiment 1—uncompressed names

The purpose of Experiment 1 was to determine whether hearing one’s own name would slow participants’ performance on a concurrent visual word categorization task more than hearing another person’s name or no name. The hypothesis was that if a participant’s attention was captured by hearing their own name, their word categorization responses would be slower than when hearing other names or no names.

## Method

### Design

The experiment was a 3 (name) × 5 (block) repeated-measures design, with three levels of name (own name, other name and no name) and five levels of block (blocks 2 through 6).

### Power analysis

A power analysis was conducted on pilot data (*n* = 12) using jpower in jamovi (The jamovi project, 2022) on word categorization latency in the presence of own name and other names. The paired-sample t-test was chosen, two-tailed, with effect size *d* = 0.7, power = 0.95 and testing at critical* p* = 0.0167 to account for three comparisons across name levels while preserving *α* = 0.05. The required sample size to find an effect, if it existed, was 37.

### Participants

A total of 39 undergraduate students from Lingnan University (20 females and 19 males) were recruited via mass emails sent to all undergraduates. They indicated their intention to participate by replying the experimenter and filling in a Google form to choose their preferred time of testing. All participants were native Cantonese speakers. They reported normal or corrected-to-normal hearing ability and vision, and had no prior participation in any of our studies investigating time-compressed speech.

Ethical approval was obtained from Lingnan University’s Research Committee (EC031/1819). Informed consent was obtained from each participant before the experiment began. Each participant received a payment of HK$100 (US$ 13) as compensation for their time.

### Apparatus

Two desktop computers (Hewlett-Packard and Lenovo) and two sets of wired headphones (Sennheiser HD206) were used, so that two participants could be run simultaneously. An application was written in PsychoPy (Peirce et al., 2019) to administer the experiment.

### Trial structure

**Fig. 1:**
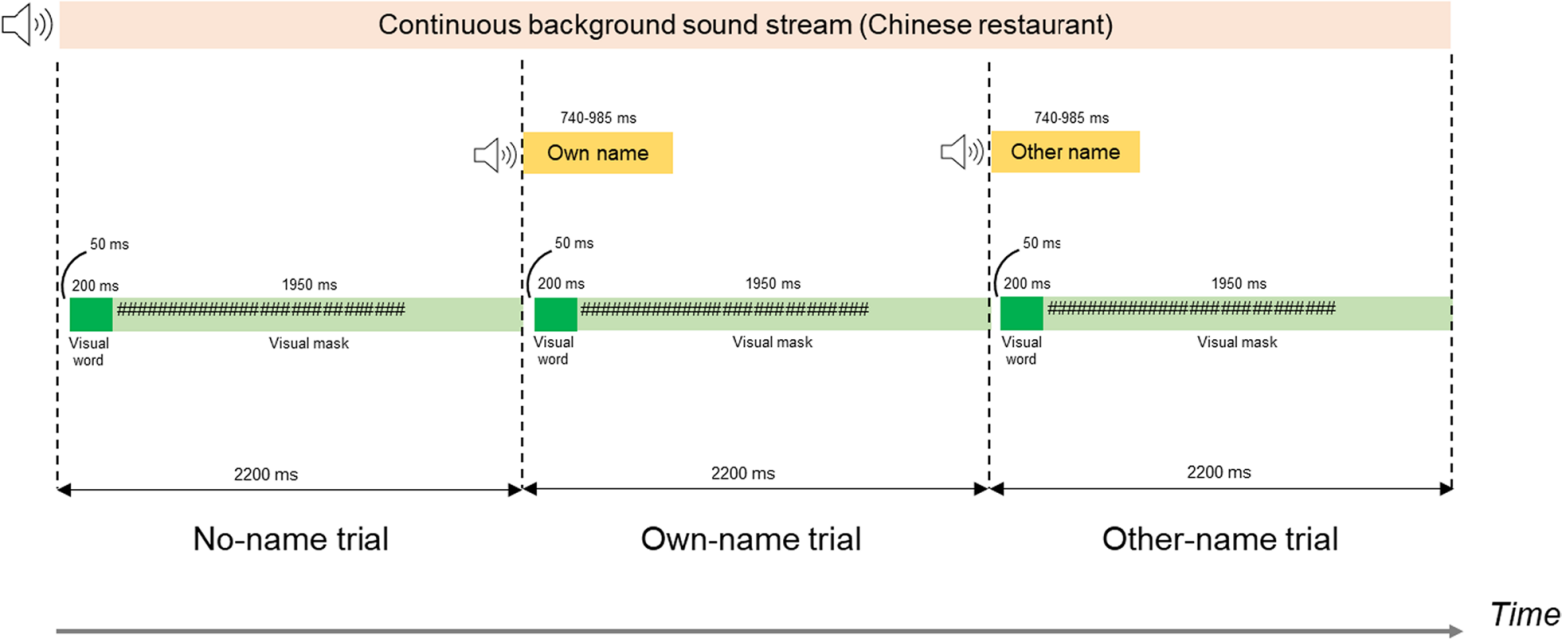
Arrangement of visual and auditory stimuli in Experiment 1 and Experiment 2. Note that trials with names would not be placed one directly after the other in the actual test blocks—the three possibilities are shown side by side here for illustrative purposes only

Figure 1 shows the arrangement of the visual and auditory stimuli. Each trial lasted 2200 ms. The visual display showed a blank screen for 50 ms, followed by a word on the screen for 200 ms (see solid green box), followed by a visual mask for 1950 ms (see pale green rectangle filled with “#” symbols). In most trials, there was no spoken name (leftmost part of figure), but a subset of trials started with either the participant’s own name (centre part of figure) or other names (rightmost part of figure). A sound stream of chatter in a busy Chinese restaurant was played continuously in the background.

### Block structure

**Fig. 2:**
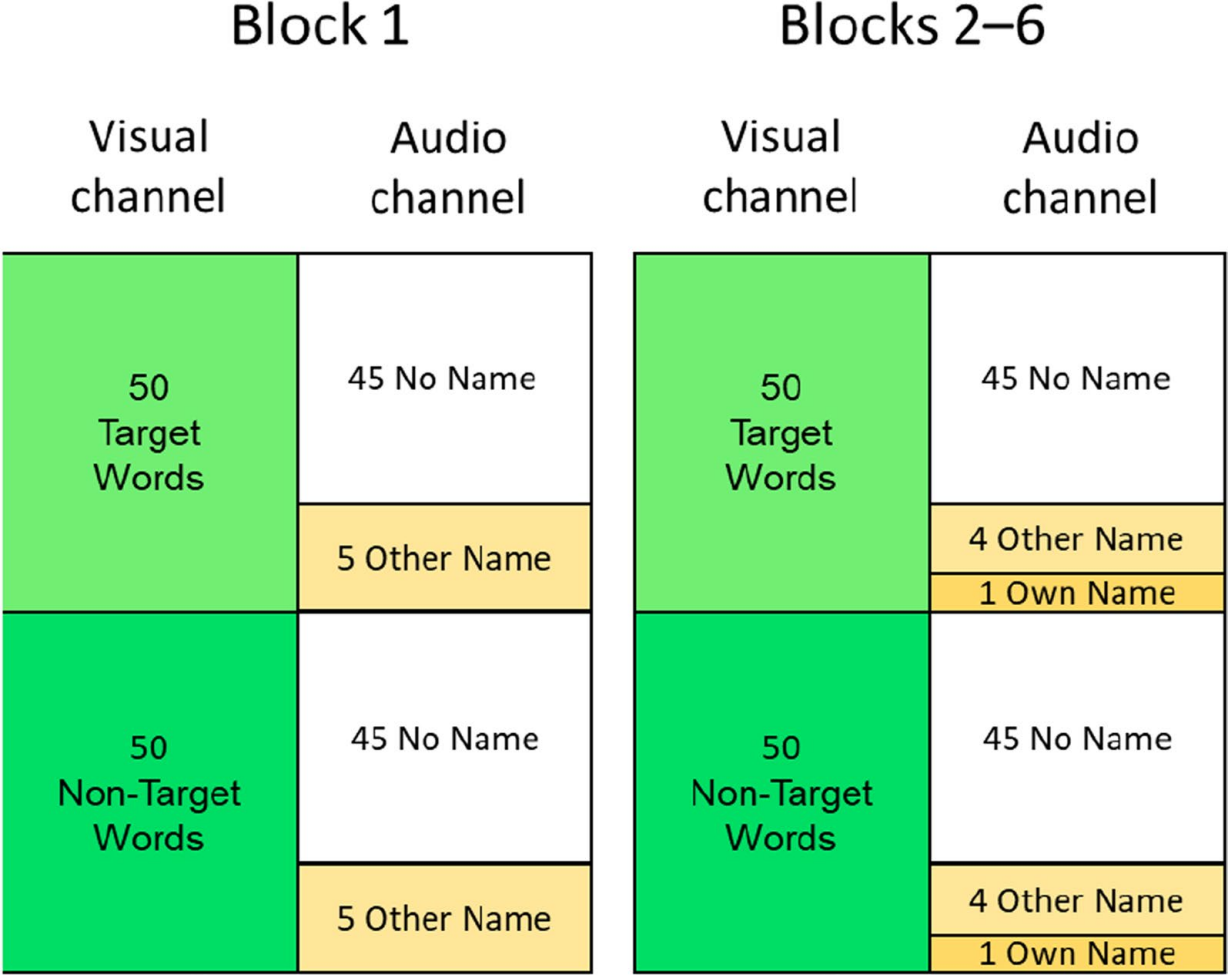
Structure of trials within each block, showing the mapping of stimuli in the visual word categorization task with no name, other name or own name in the audio channel (alongside the restaurant audio, not shown here). The post-test block used the same structure as Blocks 2–6, but participants categorized names heard as other name vs. own name rather than responding to the word categorization stimuli. The order of individual trials was randomized within each block

The test phase consisted of six blocks, each containing 100 word categorization trials (details of the test blocks are shown in Fig. 2). In each block, 50 trials displayed words in the target category and 50 trials displayed words in the non-target category. For each category of word, 5 trials contained a spoken name (own name or other name) and 45 trials contained no spoken name, so that 10 trials in each block contained spoken names.

- In Block 1, only other names were used (overall, 10 other names, with each specific other name occurring once) to avoid alerting participants early in the experiment that their own name might be played.
- In each of Blocks 2–6, other names and own name were used (overall, eight other names, with each specific other name occurring once, plus one own name, occurring twice).

The 10 trials within each block in which a spoken name was played were determined as follows. No names were placed in the first 10 trials of each block. The remaining 90 trials were then divided into 10 intervals of 9 trials with each of the ten name stimuli being randomly assigned an interval and then randomly inserted to one of the 9 trials in that assigned interval. This controlled the average spacing of names within a block, while maintaining the unpredictability of their onset.

The two own name stimuli were pseudo-randomly inserted such that the first own name occurred in the 3rd, 4th or 5th intervals (with the interval randomly determined so own name fell anywhere from the 29th to the 55th trial), and the second own name in the 8th, 9th or 10th interval (interval randomly determined so own name fell anywhere from the 74th to the 100th trial).

### Stimuli

*Visual words*. A total of 430 two-character nouns were selected from the Chinese Lexical Database (Tse et al., 2017) for the word categorization task. Forty words from the sports category were used as target words during practice. Fifty words from the animal category were used as target words during the main blocks.

*Spoken names*. Participants’ Cantonese names were obtained when they replied to the initial recruitment email. All Cantonese names have the format of surname first followed by given name. In the current study, all participants’ names had a one-character surname (e.g. 陳) and two-character given name (e.g. 智文). Each character has one syllable; therefore, all the name stimuli (own names and other names) had three syllables. Only the full name was obtained from each participant, no preferred names or nicknames were asked. Sixty other random names, all containing three syllables, were picked from an online Chinese-name generator—中文姓名產生器 v3.4 (http://www.richyli.com/name/index.asp).

The pronunciation of participants’ names were generated as speech using Microsoft Azure, an online text-to-speech generator. We used Azure’s default Cantonese pronunciation library to generate the spoken names and set Azure’s adjustable settings to the following: language set to Chinese (Cantonese, Traditional), voice set to HiuGaai (Neural), speed set to 1.00 (normal) and pitch set to 0.00 (no pitch change). The generated speech was recorded in Audacity® version 3.1.3 (https://www.audacityteam.org/) to produce a sound file for each spoken name.

*Background sound stream*. The sound stream of a busy Chinese restaurant was taken from a video on the Internet. (The link to the video is provided as supplementary materials in the OSF repository.) We extracted a 24-min block that was then cut into six four-minute sound tracks. Each of the six background sound tracks was then randomly assigned to one of the six testing blocks without repetition, so that each testing block had a unique background sound track. Therefore, each participant had a random assignment of order of the six background sound tracks over the six testing blocks.

The signal-to-noise ratio (SNR) of the spoken names to the background sound stream was in the range − 5.40 to − 5.14 dB, indicating that the spoken names were quieter than the background sound stream. Prior research indicates that speech stimuli are still intelligible in ambient noise at negative SNRs (Bradley et al., 1999). Overall sound pressure level averaged around 51dB.

### Post-test questions

After the last test block, the experimenter asked the participant the following six questions in sequence: (1) what they heard during the experiment; (2) whether they heard people talking; (3) what they heard those people talking about; (4) whether they heard any names; (5) whether they heard their own name; and (6) how many times they heard their own name.

### Procedure

Informed consent was obtained from participants before the experiment began. The phases of the experiment are described below and summarized in Table 1.

**Table 1:** Phases of the experiments. For the structure of Blocks 1 through 6, see Figs. 1 and 2

Phase of experiment	Content
*Practice (Experiment 2 only)*	Time-compressed speech training
Initial report	Listen to five three-syllable words at 25% compression and report them as heard
Compression presentation	Hear one three-syllable word at 100% (uncompressed) then at 57%, 38%, 25% compression
Familiarization with compressed words	See 10 three-syllable words while hearing them at 25% compression
Report	Listen to 10 three-syllable words (5 old, 5 new) and report each as heard
*Practice (Experiments 1 and 2)*	
Word categorization familiarization	28 trials Word categorization target category “sports” No names spoken
*Test (Experiments 1 and 2)*	
Block 1	100 trials Word categorization target category “animals” Other names spoken but no own names spoken (at appropriate compression)
Blocks 2–6	100 trials in each block Word categorization target category “animals” Other names and own names spoken (at appropriate compression)
*Post-test (Experiments 1 and 2)*	
Post-test questions	Graded series of questions probing whether own name heard Open-ended estimate of frequency of hearing own name
Name detection trial block	100 trials Visual words presented but to be ignored Discriminate own name vs. other name (at appropriate compression)

*Practice.* Participants completed a practice block of 28 trials (14 target trials and 14 non-target trials) to familiarize them with the word categorization task. They were asked to place their right index finger over the “m” key and left index finger over the “z” key on the keyboard. Participants were told that the target word category was “sports”. They pressed “m” when a target word appeared and “z” when a non-target word appeared. Participants were instructed to perform the word categorization to the best of their ability and to ignore the sounds. Participants heard the background soundtrack through headphones, but no spoken names were presented and no feedback about performance was given during practice.

*Test*. The test phase consisted of six blocks of 100 word categorization trials each, as illustrated in Figs. 1 and 2 and described in the Block Structure subsection. Participants were told that the target word category was “animals”. The key mappings for responding were the same as for the Practice phrase. Participants again wore headphones and were instructed to perform the word categorization to the best of their ability and to ignore the sounds. They were not told that names would be presented. A two-minute break was allowed between each block of trials.

*Post-test questions*. The experimenter asked the participants the post-test questions.

*Post-test name detection block*. In a final block with the same visual and auditory stimuli as blocks 2–6, participants were instructed not to respond to the words appearing on the screen but instead to listen for spoken names. They were asked to press the “m” key if they heard their own name and the “z” key if they heard any other name. Finally, participants were debriefed by the experimenter.

A maximum of two participants were tested simultaneously in each experimental session. Each participant was in a separate room next to each other. The experiment was conducted in Cantonese and lasted approximately 60 min.

### Primary and secondary outcomes

The primary outcome was the effect of name condition on word categorization latency, measured from the start of the visual presentation of the word until the participant’s keypress response. Secondary outcomes included (a) the effect of block on word categorization latency to assess whether there was habituation to hearing one’s own name (Röer et al., 2013); (b) word categorization accuracy to check whether participants were adequately performing the task; (c) self-report responses in the post-test questionnaire to check whether participants heard their own name; and (d) name detection accuracy and latency in the post-test name detection block to assess whether participants heard their own name and whether there was a difference in how fast they responded to own name versus other name.

### Statistical analysis plan

*Data screening*. In preparation for calculating latency of word categorization, we eliminated certain responses. First, we eliminated responses that were implausibly fast (< 130 ms) or slow (> 2200 ms). The lower limit of 130 ms was selected as the minimal reaction time required in two-choice reaction time tasks (Card et al., 1983), and the upper limit was the time elapsed until the next trial began. Second, we eliminated responses if a valid key press (“z” or “m”) was not recorded. Third, we eliminated *all* data from any participant whose no name word categorization accuracy was two standard deviations above or below the mean, reasoning that they were either not following the task instructions or not paying enough attention to the task.

Data screening was also performed on the post-test name detection block, where participants were asked to discriminate own name from other names for the 10 name presentations within the block. Data from participants who made more than 10 key presses during the no name trials were excluded, because it suggested that they were still responding to the word categorization task rather than discriminating other name vs. own name.

*Test assumptions*. Visual inspection of Q–Q plots determined whether residuals were normally distributed; if not, log_10_ transformations would be attempted. If the same statistical pattern was obtained with the transformed and untransformed data, descriptive and graphical information from the untransformed data would be reported. Greenhouse–Geisser corrections were used if sphericity was violated. If data transformation was unsuccessful, nonparametric methods were used.

*Tests of hypotheses*. A 3 (name) × 5 (block) repeated ANOVA was planned to test the primary outcome of name condition on word categorization latency. Three post hoc comparisons between paired name conditions were done with a Bonferroni correction (adjusted critical *p* = 0.0167). A similar ANOVA let us test the secondary outcome of effect of block on latency. Any significant interaction between name and block would be followed up with an analysis of trends in latency change across blocks for each name condition.

An exploratory analysis was performed for word categorization accuracy, using a Friedman ANOVA followed by Durbin–Conover pairwise comparisons. For the self-report of hearing own name, an error score was calculated for each participant for their estimate of how frequently they heard their own name, using the following formula, where a score of 0 indicates no deviation from perfect accuracy of 10.

$${\text{Error score = Reported number of times heard own name{-}10}}$$

For the post-test name detection block, a paired-sample *t*-test was planned to assess difference in latency for detecting own name and other name. An exploratory test of post-test name detection accuracy used the percentage of correct identification of own name or other names. All statistical tests were carried out using jamovi.

## Results

Data were obtained from 39 enrolled participants (20 females and 19 males). After data screening, two participants were identified as having accuracy more than two standard deviations below the average accuracy for the group (*M* = 95.0%, *SD* = 6.4%) so their data were excluded. The final sample was 37 participants. After excluding trials with no response, the dataset comprised 22,033 trials (99.25% of all trials).

### Primary outcome

*Effect of name on word categorization latency*. Word categorization latency data were subjected to a log_10_ transformation, and a 3 (name condition) × 5 (blocks) repeated ANOVA was conducted. The main effect of name was significant (see Table 2). Post hoc comparisons indicate that the presence of own name resulted in significantly slower word categorization than the presence of other name, *t*(36) = 6.43, *p* < 0.001, or no name, *t*(36) = 6.42, *p* < 0.001, but there was no significant difference in latency between other name and no name, *t*(36) = 0.61, *p* = 0.54.

**Table 2:** Results of Experiment 1 with uncompressed names (100% of original duration). Descriptive statistics are mean (standard error of the mean) or median [lower quartile, upper quartile]

Measure	Own name	Other name	No name	Effect of name
Word categorization latency (ms) *M* (*SE*) (*n* = 37)	772 (23)	669 (16)	662 (16)	*F*(1.22, 44.09) = 38.50, *p* < .001, *η*^*2*^_*p*_ = 0.517
Word categorization accuracy *Mdn* [*LQ, UQ*] (*n* = 37)	100% [90–100]	98% [96–100]	98% [96–99]	
Post-test name detection latency (ms) *Mdn* [*LQ*, *UQ*] (*n* = 31)	901 [781–1069]	1085 [975–1151]	–	*W* = 452, *p* < .001, *r*_*rb*_ = 0.823
Post-test name detection accuracy *Mdn* [*LQ, UQ*] (*n* = 31)	100% [100–100]	100% [100–100]	–	

### Secondary outcomes

**Fig. 3:**
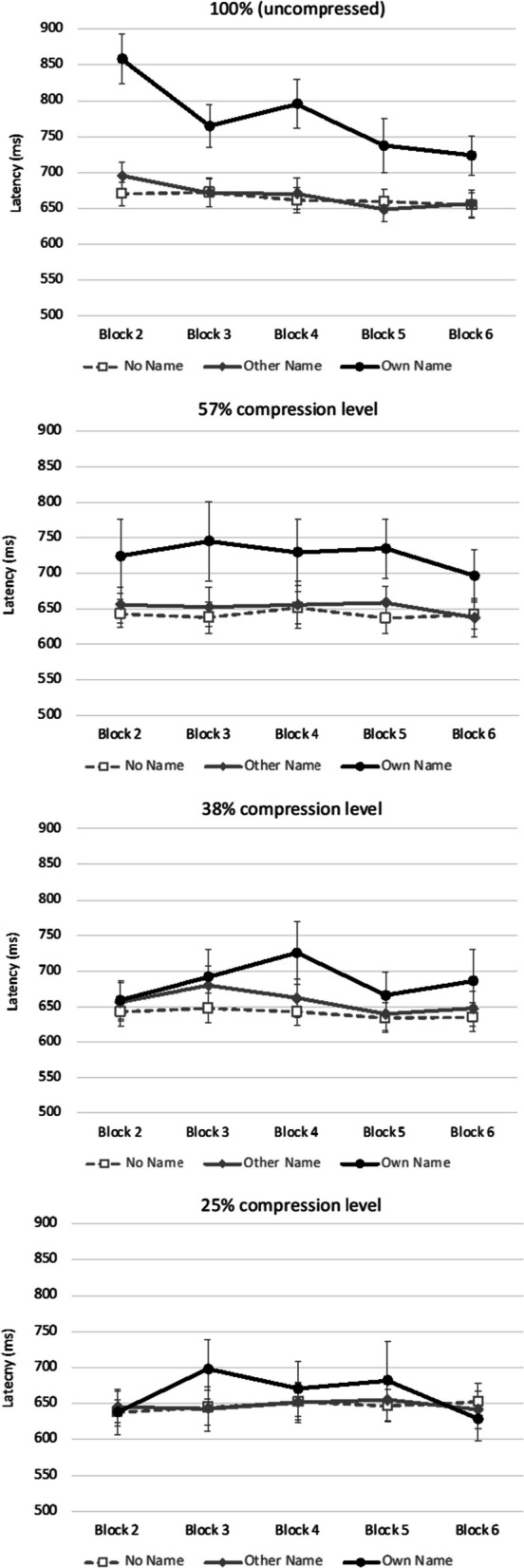
Block by block latency of response to visual word categorization for Experiment 1 (100% uncompressed: top graph) and Experiment 2 (57%, 38% and 25% compression: lower graphs). Error bars are standard error of the mean

*Effect of block on word categorization latency*. The top panel in Fig. 3 shows the untransformed latency data for each of the name conditions from blocks 2 to 6 (there were no own name trials in block 1). The main effect of block was significant, *F*(2.95, 106.19) = 7.04, *p* < 0.001, *η*^*2*^_*p*_ = 0.164, as was the interaction of name condition and block, *F*(4.58, 164.81) = 2.77, *p* = 0.023, *η*^*2*^_*p*_ = 0.071. To evaluate the effect of blocks on latency, we obtained the linear regression slope of latency on blocks for each participant in each name condition. The slope represents the averaged change in latency as a participant progressed from one block to another, where a negative value represents faster responses over blocks, and where tests are one-sample t tests against a slope of 0 ms/block.

Latency in ms became faster over blocks for own name (*M* = − 29.67, *SD* = 43.36, 95% C.I. = [− 44.13, − 15.22], *t*(36) =  − 4.16, *p* = 0.0002, Cohen’s *d* = 0.68). Latency also became faster over blocks for other name (*M* = − 10.10, *SD* = 30.20, 95% C.I. = [ − 20.17, − 0.03], *t*(36) =  − 2.03, *p* = 0.0494, Cohen’s *d* = 0.33). However, latency remained fairly constant over blocks for no name (*M* = − 4.25, *SD* = 16.98, 95% C.I. = [ − 9.91, -1.41], *t*(36) =  − 1.52, *p* = 0.14, Cohen’s *d* = 0.25).

*Word categorization accuracy*. Accuracy scores for the word categorization task are shown in Table 2. Accuracy for own name, other name and no name was at ceiling levels of 100%, 98% and 98%, respectively; therefore, only descriptive statistics are provided.

*Self-report of hearing own name*. In the post-test questionnaire, all 37 participants reported hearing their own name (see Table 3). Table 4 shows that 11 (30%) participants perfectly estimated own name frequency, 15 (40%) participants underestimated it (score < 0), and 11 (30%) participants overestimated it (score > 0).

**Table 3:** Responses to the post-test question “Did you hear your own name?” by compression level. Entries are number and percentage of participants who reported hearing their own name at least once

Compression level	Did you hear your own name?		
	No	Yes	Total
*Experiment 1*			
100% (uncompressed)	0 (0%)	37 (100%)	*n* = 37
*Experiment 2*			
57%	0 (0%)	24 (100%)	*n* = 24
38%	4 (17%)	20 (83%)	*n* = 24
25%	19 (83%)	4 (17%)	*n* = 23

**Table 4:** Error scores (-10 to 10) for participants’ estimates of the number of times they heard their own name during Experiment 1 and Experiment 2

	Underestimate										Accurate									Overestimate	
	− 10	− 9	− 8	− 7	− 6	− 5	− 4	− 3	− 2	− 1	0	1	2	3	4	5	6	7	8	9	10
*Experiment 1*																					
100% (n = 37)	–	–	–	1	1	2	6	1	2	2	***11***	1	7	1	–	2	–	–	–	–	–
*Experiment 2*																					
57% (n = 24)	–	–	–	–	2	2	3	–	2	–	***6***	2	3	–	–	–	–	–	1	–	3
38% (n = 24)	**4**	1	1	2	1	2	*1*	1	2	1	3	–	3	1	–	1	–	–	–	–	–
25% (n = 23)	***19***	1	–	1	–	–	–	–	1	–	1	–	–	–	–	–	–	–	–	–	–

Values of 0 represent perfect accuracy, positive values are overestimates, and negative values are underestimates; − 10 indicates that participants did not hear their own name on any of the 10 presentations of own name. Entries are the count of participants with a specific error score. Values in italics indicate medians, those in bold indicate modes, and those in bold italics indicate that median and mode are the same value

*Post-test name detection latency*. After data screening was applied to latency data from the name detection block, six participants’ data were excluded, giving a total analysable sample of 31. Participants were faster at detecting their own name than other names (see Table 2).

*Post-test name detection accuracy*. In the post-test block when participants were instructed to pay attention to the auditory presentations of the names, they could discriminate their own names from other names with accuracy at ceiling levels of 100%. Therefore, only descriptive statistics are presented (see Table 2).

## Discussion

As predicted, participants’ word categorization was slower by over 100 ms when their own name was heard, but not when other names were heard or when there was no accompanying name. This finding can be accounted for by the specific attentional capture mechanism in the duplex account of auditory distraction, which suggests that when stimuli have similar physical properties, stimuli with specific relevance to the listener would be more distracting than those without such relevance (Hughes, 2014; Hughes et al., 2005).

The relatively slower responding with own name lessened over blocks suggests that participants were habituating to the effect of their own name (Röer et al., 2013). Habituation was also observed for other name and this is consistent with findings that show distraction by irrelevant sounds can be reduced by repeated exposure (Bell et al., 2012; Röer et al., 2014). The difference in the effect sizes of habituation for own name (Cohen’s *d* = 0.68) and other name (Cohen’s *d* = 0.33) was about twofold, suggesting that the rate of habituation of own name was faster than other name. This is likely because there was more initial slowing of responses caused by the distraction from own name (~ 850 ms) than other name (~ 700 ms) in the early trial blocks. This further supports own name has a higher attentional capture potential than other name.

Word categorization was highly accurate in all name conditions, with medians at 98% or above. Any differences in the descriptive statistics most likely reflect the unusual underlying distributions and should probably not be given too much importance.

All participants reported hearing their own name during the main blocks, despite the presence of background noise and their focus on the word categorization task. At face value, this seems to be in contrast to classic dichotic listening experiments, in which only about 30% of participants recalled hearing their own name in the unattended ear (Moray, 1959; Röer & Cowan, 2021; Wood & Cowan, 1995). However, the present experiment offered 10 opportunities to hear own name, compared with the more limited opportunities in most previous research. For example, in Moray (1959), each participant only received three opportunities to hear their own names (Experiment 2). Furthermore, the high number of participants reporting hearing their own name is not uncommon. In Röer et al. (2013), 53 out of 55 (96%) participants reported they had heard their own name after the experiment (p. 929).

Although the multi-modal nature of the current experiment may have left participants with enough attentional resources to detect their own name (Wickens, 2008), 70% of participants either underestimated or overestimated how often their own name occurred. This is likely a problem of memory or judgment rather than a problem of attention or perception. It is unlikely that participants in the present experiment study were using their spared resources, if any, to monitor the ignored channel. If participants were actively monitoring for names in the background then, according to the irrelevant sound effect, any names should result in some level of slowing of word categorisation performance. However, the current results suggest that other names were like no name which showed no slowing of word categorisation; only own name resulted in slowing.

In the post-test name detection block, participants were faster at detecting their own name than other names. Participants’ strong high-level lexical representation of own name may have helped them detect it more rapidly than other names (Ahissar et al., 2009; Nahum et al., 2008).

## Experiment 2—time-compressed names

Experiment 1 showed that participants’ word categorization performance was slower when they heard their own name than when they heard some other name. The purpose of Experiment 2 was to examine whether the effect would persist when own and other names were time-compressed. It is known that speech becomes hard to identify when compressed to less than 30% of its original duration (Sabic & Chen, 2016). However, it is unclear how much different degrees of compression may remove acoustic cues from personally relevant verbal stimuli that would otherwise trigger high-level lexical representations and therefore capture attention (Banai & Lavner, 2012; Srbinovska et al., 2021).

Accordingly, we tested the effect of the name condition on word categorization with the names at different levels of compression. We were interested to discover whether even strongly time-compressed own name would still capture attention sufficiently to slow word categorization, or whether there would be a level of compression at which own name ceased to capture attention.

## Method

### Design

The experiment was a 3 (name condition) × 5 (block) × 3 (compression level) mixed design with within-subjects factors of name (no name, other name and own name) and block (Block 2–6), and a between-subjects factor of compression level (57%, 38%, 25% of original duration).

### Power analysis

A pilot study (*n* = 6) testing the 38% compression-level condition was carried out to estimate the required sample size. A power analysis was conducted to compare latency from the own name and other name conditions, two-tailed, with effect size, Cohen’s *d* = 0.7, power = 0.8 and testing at critical *p* = 0.025, to retain *α* = 0.05 for two comparisons: own name vs. other name and own name vs. no name. The recommended sample size for each compression condition to find an effect if it existed was 23; accordingly, we sought 25 participants in each condition.

### Participants

A total of 75 undergraduate students from Lingnan University (44 females and 31 males) were recruited as participants with the same selection criteria as Experiment 1. The 38% and 25% conditions were carried out first and participants were randomly assigned to one of the conditions. The 57% condition was carried out a month later, but its participants were from the same semester cohort and were run under the same experimental conditions by the same experimenter as the first two conditions. Each participant received a payment of HK$100 (US$13) as compensation for participation.

### Apparatus

The same apparatus as in Experiment 1 was used.

### Stimuli

The time-compressed names were created using Audacity via the “Sliding Stretch” effect to compress the spoken name stimuli. Sliding Stretch uses Subband Sinusoidal Modeling Synthesis (SBSMS), a high-quality compression algorithm that performs a spectral analysis of the original sound and compresses based on the output of the analysis (https://github.com/claytonotey/libsbsms). SBSMS has the benefit of producing less acoustic artefact in the compressed output than alternative methods such as Synchronous Overlap–Add (SOLA) methods. Three compression conditions were generated, with each name compressed to 57%, 38% and 25% of its original duration.^1^ For example, each name from the 57% condition lasted 57% of its original uncompressed duration.

### Procedure

The procedure was similar to the procedure of Experiment 1 except that a compressed speech training phase was added before the practice phase—see Table 1. The training phase had four steps.

1. Participants listened to a list of five three-syllable Chinese words (紅綠燈 “traffic lights”; 小提琴 “violin”; 洗頭水 “shampoo”; 奶油多 “condensed milk toast”; and 信用卡 “credit card”) in time-compressed format at the most extreme compression level, 25%. Participants were asked to report what the words were.
2. A three-syllable word was presented to participants with successively strong compression levels, from 100% (uncompressed), to 57%, 38% and 25% of original duration. The purpose was to have participants experience how all three compression levels changed how a word sounded. No response was required from the participants.
3. A list of 10 new three-syllable words was presented visually to participants on a laptop, and they listened to the words played at the 25% compression level while looking at the words on screen. No response was required. This step provided participants an opportunity to hear the time-compressed speech and see its written form at the same time.
4. Ten spoken words (five from the step 3 list and five new) were played to participants at 25% compression, and they were asked to report what the words were. This step tested whether participants had learnt the previously heard words and could identify the new ones.

Steps 3 and 4 trained and tested participants on the 25% compression level assuming that if they could identify words at the most extreme compression level, then they should have no problem understanding content with the weaker compression levels.

The remainder of the Experiment 2 proceeded as for Experiment 1.

### Primary and secondary outcomes

The primary and secondary outcomes were as in Experiment 1.

### Statistical analysis plan

*Data screening*. The same data screening procedures were used as in Experiment 1.

*Test assumptions*. Statistical assumptions and procedures to address violations of assumptions were as in Experiment 1. Nonparametric tests were planned if there was no suitable or effective data transformation method for the data.

*Test of hypothesis*. A 3 (name) × 5 (block) × 3 (compression) mixed-effects ANOVA was planned to analyse the effects of name condition, block and compression level on word categorization latency. Post hoc comparisons with any significant main effect of name were adjusted with Bonferroni corrections, so that the critical value of *p* was 0.0167 to retain alpha at 0.05. Any significant interaction between name and compression was followed with one-way repeated ANOVAs, one for each compression level, to test for an effect of name condition at that level. Any significant effect of name at that level was followed with a paired-sample t-test comparing own name vs. other name and own name vs. no name (adjusted critical *p* = 0.025). The statistical plan for the secondary outcomes followed a similar process as in Experiment 1.

## Results

After data screening, four participants had an average word categorization accuracy more than two standard deviations below the mean and were removed from analysis (two from 25%, one from 38% and one from 57% condition). After excluding trials with no response, a total of 42,370 trials (99.46% of all trials) from 71 participants were included in the following analyses. Most descriptive and inferential results are in Tables 3, 4 and 5; further results are in the text below.

**Table 5:** Results of Experiment 2: Word categorization performance (blocks 2–6) and name detection performance (post-test block) for time-compressed names, according to compression level

Measure compression (%)	Own name	Other name	No name	Effect of name	Effect of compression	Interaction
*Word categorization latency (ms) M (SE)*						
57% (*n* = 24)	729 (37)	653 (23)	644 (20)	*F*(1.35, 327.05) = 12.73, *p* < .001, *η*^*2*^_*p*_ = 0.160	*F*(2, 67) = 0.06, *p* = .944, *η*^*2*^_*p*_ = 0.002	*F*(2.70, 327.05) = 3.27, *p* = .029, *η*^*2*^_*p*_. = 0.089
38% (*n* = 24)	686 (28)	657 (21)	642 (19)			
25% (*n* = 23)	664 (30)	650 (23)	647 (21)			
*Word categorization accuracy Mdn [LQ, UQ]*						
57% (*n* = 24)	100% [100–100]	98% [96–100]	98% [95–99]		χ^*2*^(2) = 1.11 *p* = .575	–
38% (*n* = 24)	100% [90–100]	98% [96–98]	98% [95–99]			
25% (*n* = 23)	100% [100–100]	98% [96–98]	97% [97–98]			
*Post-test name detection latency (ms) M (SE)*						
57% (*n* = 23)	804 (34)	938 (40)	–	*F*(1, 59) = 32.70, *p* < .001, *η*^*2*^_*p*_ = 0.357	*F*(2, 59) = 2.41, *p* = .099, *η*^*2*^_*p*_ = 0.076	*F*(2, 59) = 0.46, *p* = .636, *η*^*2*^_*p*_ = 0.015
38% (*n* = 23)	825 (42)	981 (41)	–			
25% (*n* = 16)	992 (89)	1113 (60)	–			
*Post-test name detection accuracy Mdn [LQ, UQ]*						
57% (*n* = 23)	100% [100–100]	100% [88–100]	–		χ^*2*^(2) = 24.2 *p* < .001	*–*
38% (*n* = 24)	100% [100–100]	88% [88–100]	–			
25% (*n* = 20)	50% [0–100]	63% [50–88]	–			

Descriptive statistics are mean (standard error of the mean) or median [lower quartile, upper quartile]

There were five participants (one in the 38% condition and four in the 25% condition) who did not press any key when their own name was played during the name detection block. As a result, their name detection accuracy was taken to be 0%, but they had to be excluded in the name detection latency analysis because their responses were missing. This lead to the discrepancy in the sample sizes between post-test name detection latency and accuracy results

### Primary outcome

*Effects of name and compression on word categorization latency*. The mixed-effects ANOVA on the log_10_-transformed latency data revealed a significant main effect of name (see Table 5 for statistics). Post hoc comparisons revealed significantly slower overall word categorization for own name, (*M* = 693 ms, *SE* = 18.6) than for other name (*M* = 653 ms, *SE* = 12.6), *t*(67) = 3.37, *p* = 0.001 and no name (*M* = 644 ms, *SE* = 11.4), *t*(67) = 4.00, *p* < 0.001, but no significant difference in word categorization latency between other name and no name, *t*(67) = 1.77, *p* = 0.081.

The main effect of compression on word categorization latency was not significant, but the interaction of name and compression was significant, as predicted (see Table 5 for means within compression levels). Within the 57% compression level, the effect of name was significant (*F*(2, 46) = 16.90, *p* < 0.001, *η*^*2*^_*p*_ = 0.423), showing slower word categorization with own name than with other name, *t*(23) = 4.10, *p* < 0.001, or with no name, *t*(23) = 4.31, *p* < 0.001. Within the 38% compression level also, the effect of name was significant (*F*(2, 46) = 5.11, *p* = 0.01, *η*^*2*^_*p*_ = 0.182), showing slower word categorization with own name than with no name, *t*(23) = 2.70, *p* = 0.013, but there was no difference between own name and other name, *t*(23) = 1.80, *p* = 0.085. Within the 25% compression level, the effect of name was not significant (*F*(2, 44) = 0.41, *p* = 0.668, *η*^*2*^_*p*_ = 0.018).

### Secondary outcomes

*Effect of block on word categorization latency*. The above mixed-effects ANOVA also showed that the main effect of block was not significant, *F*(3.51, 235.36) = 1.11, *p* = 0.349, *η*^*2*^_*p*_ = 0.016 (see Fig. 3). In contrast to Experiment 1, the interaction between name and block did not reach significance, *F*(4.88, 327.05) = 0.79, *p* = 0.559, *η*^*2*^_*p*_ = 0.012.

*Word categorization accuracy*. Table 5 shows the word categorization accuracy for each compression level across name conditions and associated statistics. Accuracy was high in all conditions, suggesting near-ceiling performance. Only descriptive statistics are presented.

*Self-report of hearing own name*. Table 4 summarizes participants’ responses to Question 5 in the post-test questionnaire. Evidently, the stronger the compression level, the fewer participants reported hearing their own name. With 57% compression, error scores for estimating the number of times own name were heard were distributed around 0; with 38% compression, most error scores were less than 0 suggesting underestimates; and with 25% compression, most participants did not hear their name at all (error score of -10).

*Post-test name detection latency*. When participants discriminated their own name from other names in the post-test block, latency could be assessed for 67 participants after data screening. A 2 (name) × 3 (compression) mixed ANOVA on log_10_-transformed name detection latency data revealed a significant main effect of name (see Table 5). Participants responded faster to their own name than to other names. However, neither the main effect of compression nor the interaction of name and compression level was significant.

Additional analysis was conducted to examine the name detection latency data in terms of correct and incorrect identification. The overall result suggests that there was no significant difference in latency between correct and incorrect responses. Details of the analysis are included in the supplementary materials.

*Post-test name detection accuracy*. Name detection accuracy for identifying other names and own name in each compression level is summarized as descriptive statistics in Table 5. A Kruskal–Wallis test showed that post-test name detection accuracy dropped as compression intensified, with lower accuracy with 25% compression (*Mdn* = 60%. *IQR* = 47–80%) than with 38% compression (*Mdn* = 90%. *IQR* = 80–100%, *p* < 0.001) or with 57% compression (*Mdn* = 100%. *IQR* = 90–100%, *p* < 0.001).

## Discussion

Participants’ word categorization accuracy was near ceiling in all three compression levels, indicating that participants were focused appropriately on the word categorization task. Overall, word categorization was significantly slower in the presence of time-compressed own name than in the presence of time-compressed other names or no name at all. However, name condition and compression interacted. At the 57% compression level, word categorization was slower in the presence of own name than other name. For the 38% compression level, word categorization with own name just failed to be significantly slower than with other name, but it was significantly slower than with no name. For the 25% compression level, there was no difference in latency across name conditions. Clearly, the attentional capture potential of personally relevant compressed speech reduces as the speech becomes more time-compressed. Supporting this, the post-test name detection accuracy results show that when names were compressed to the 25% level, and participants were asked to focus entirely on discriminating their own name from other name, they had considerable difficulty in doing so. If own name cannot be distinguished from other names under focal attention conditions, it is unsurprising that own name should cease to attract attention and slow responding on a timeshared task.

Word categorization latencies provided no evidence that participants habituated to the effect of their own name across blocks for any compression level. This occurred despite the fact that for the 57% compression group, name frequency estimates were broadly comparable to the results for participants in Experiment 1 who experienced no compression, and their post-test name detection accuracies were high. It seems that at the relatively mild 57% level of compression, own name engaged participants’ attention enough to slow word categorization, but less than required for habituation to set in.

## General discussion

The purpose of the two experiments was to determine whether personally relevant time-compressed speech phrases (own name) inserted into an unattended auditory channel would slow responses to a concurrent visually presented word categorization task more than personally irrelevant speech phrases (other name) would. The experiments were a first step towards testing whether professionally relevant time-compressed speech, such as information about a deteriorating patient, would draw attention from other tasks as reliably as professionally relevant uncompressed speech might do. As an initial test of the concept we used the participant’s own name, given the repeated evidence of its success in capturing attention (Conway et al., 2001; Moray, 1959; Röer & Cowan, 2021; Röer et al., 2013; Wood & Cowan, 1995). After performing a partial replication in Experiment 1 with uncompressed versions of the name stimuli, we tested the effect in Experiment 2 with compressed versions of the name stimuli.

Both experiments showed that the presence of the participant’s own name in the unattended background sound slowed word categorization latency more than the presence of other names. Moreover, in both experiments, other names did not slow word categorization compared with no names, indicating that the slowdown with own names was not simply due to auditory distraction. However, the strength of the slowing depended on the compression level; the significant interaction between name and compression level obtained in Experiment 2 suggests that the slowing diminishes as speech compression becomes more extreme. The slowing effect was strongest when the spoken names were compressed at the 57% compression level, weaker at the 38% level and non-existent at the 25% level. The results can be readily explained as auditory distraction due to *specific attentional capture* (Hughes, 2014). Word categorization latency slows because participants’ focal attention is diverted by the personally relevant content of the sound. The effect disappeared at the 25% compression level probably because the names were so distorted that when the participant’s focal attention was elsewhere, own name was not recognized as such, and so was incapable of capturing attention.

The habituation that we found in response to uncompressed own name in Experiment 1 is similar to findings by Röer et al. (2013), who showed that the disruptive effect of hearing ones’ own name on serial memory recall decreased as participants encountered more instances of own name, but not other names. Response to hearing other names also showed habituation, but it was smaller in magnitude. This could be because our measure (latency in a choice reaction task) is more sensitive than Roer et al.’s (accuracy in serial recall task) to detect habituation. Importantly, habituation of other name is consistent with findings that irrelevant sounds (e.g. normal and reversed speech, or music) become less disruptive over time (Bell et al., 2012; Röer et al., 2014). This provides support for the theoretical position that proposes attention is required for maintaining information in working memory (Röer et al., 2014) and explaining distraction caused by irrelevant sounds (Bell et al., 2012) as opposed to interference purely caused by non-attentional processes such as acoustic changes. The habituation to relevant and irrelevant stimuli in the current study also provides support for the attentional component of the duplex-mechanism account (Hughes, 2014). Habituation to relevant stimuli (e.g. own name) and irrelevant stimuli (e.g. other name) could be accounted for by the specific and aspecific attentional capture mechanisms, respectively.

However, in Experiment 2, no habituation to own name was found in any of the compression levels possibly because the compressed own name did not reach a detection threshold when focal attention was elsewhere. Although attentional capture was observed in the 57% compression level, it also did not show habituation. It might be that compressed own name need longer time (i.e. more trial blocks) for participants to get habituated. Nonetheless, both experiments show that in the post-test name detection block, participants detected their own name faster than other names when focused on name detection, although the differences were only significant at the 57% compression level.

There are several limitations in the current study. First, the compressed speech training period in Experiment 2 was relatively short, compared with previous experiments (Sanderson et al., 2019). The fact that we did not find attentional capture with the 37% and 25% compression levels could potentially be because participants were still relatively unfamiliar with the sound of highly compressed speech. Prior research suggests that stable, generalizable learning of time-compressed speech only occurs after multi-session practice (Gabay et al., 2017).

Second, it is unclear whether personally relevant time-compressed speech would demonstrate attentional capture when focal attention is directed at other *auditory* stimuli, rather than to visual stimuli. Further research involving a simultaneous listening task would help establish the effect with better control.

Third, our participants were asked to ignore the background audio, but they were not assured that they were not going to be tested about it (Jones & Macken, 1993); therefore, participants could have been attending to the background audio. Nevertheless, participants in both experiments exhibited near-ceiling performance in the word categorization task, which suggests that their focal attention was not on the background audio.

Fourth, it may be that other algorithms for time-compressed speech are more effective at preserving phonemic features important for understanding own name. Although the SBSMS algorithm is of high quality and computationally intensive, using the frequency domain, algorithms in the OLA family that use the temporal domain may be more effective.

Fifth, the names were presented 50 ms earlier than the visual word. If participants could process the name from its first 50 ms before seeing the word, then attentional capture from a name might not be at the preattentive level. However, the 50-ms delay was perceptually negligible from pilot reports. Moreover, (a) the delay was present for both own name and other names, denying any time advantage to one condition over the other, yet responding slowed only for own name, and (b) word categorisation latency was not significantly different across other names and no names.

Our work on time-compressed speech representing clinical vital signs has shown that participants, despite having no clinical training or knowledge, could accurately identify time-compressed speech phrases representing patient’s blood oxygen and heart rate levels (e.g. low oxygen saturation and high heart rate) in Cantonese (Li et al., 2017) and English (Sanderson et al., 2019). A next step is to test whether compressed speech indicating patient health deterioration (e.g. low blood oxygenation and high heart rate) would reliably attract health professionals’ attention in the preattentive manner recommended by (Woods, 1995).

When using compressed speech for patient monitoring, a desirable feature would be to broadcast normal-level vital signs (e.g. normal blood oxygenation and normal heart rate) continuously (e.g. every 30 s) to inform the listener that all is well. The continuous message would become background sound to the listener without demanding their focal attention, like the busy Chinese restaurant sound stream used in the current study. When there is deterioration in the vital signs, an abnormal-level vital sign compressed speech (e.g. low blood oxygenation and high heart rate) would break through the listener’s focal attention; like hearing one’s own name. This has important practical implications for using time-compressed speech for patient monitoring, especially in multitasking. Future research would be to establish whether personally or professionally relevant compressed speech varies in its ability to capture attention according to factors such as increasing task demand of the focal task (Hughes et al., 2013), expectancy (Vachon et al., 2012) and working memory capacity (Conway et al., 2001). Outcomes will have practical as well as theoretical implications and will contribute to the duplex account of attentional capture.

## Conclusion

The distraction effect from personally relevant auditory stimuli from the current experiments is a demonstration of *specific attentional capture* as proposed by the duplex account of auditory distraction. Our findings suggest that the attentional capturing effect can be extended to personally relevant time-compressed speech, but level of capture depends on the level of compression. Our current approach is to view auditory distraction as a cognitive phenomenon that can be exploited to attract attention of workers performing other tasks. Further research is required to test professionally relevant time-compressed speech with domain experts before it could be used in industry application.

## Data Availability

The studies were not preregistered. The raw data or datasets generated and/or analysed during the current study and various supplementary materials are available in the OSF repository, https://osf.io/mrfyw/.

## Footnotes

1 The original compression rates that were aimed for were 50%, 25% and 15%. However, upon the completion of data collection we discovered that our “Sliding Stretch” effect settings had resulted in compressed speech stimuli with the reported compression rates instead. Further details are available on request.

## References

[CR1] Ahissar M, Nahum M, Nelken I, Hochstein S (2009). Reverse hierarchies and sensory learning. Philosophical Transactions of the Royal Society B-Biological Sciences.

[CR2] Banai K, Lavner Y (2012). Perceptual learning of time-compressed speech: More than rapid adaptation. PLoS ONE.

[CR3] Bell R, Röer JP, Dentale S, Buchner A (2012). Habituation of the irrelevant sound effect: Evidence for an attentional theory of short-term memory disruption. Journal of Experimental Psychology: Learning, Memory, and Cognition.

[CR4] Bradley JS, Reich RD, Norcross SG (1999). On the combined effects of signal-to-noise ratio and room acoustics on speech intelligibility. Journal of the Acoustical Society of America.

[CR5] Card SK, Moran TP, Newell A (1983). The Psychology of Human-Computer Interaction.

[CR6] Cherry EC (1953). Some experiments on the recognition of speech, with one and with two ears. Journal of the Acoustical Society of America.

[CR7] Conway ARA, Cowan N, Bunting MF (2001). The cocktail party phenomenon revisited: The importance of working memory capacity. Psychonomic Bulletin & Review.

[CR8] Dalton P, Hughes RW (2014). Auditory attentional capture: Implicit and explicit approaches. Psychological Research Psychologische Forschung.

[CR9] Deschamps M-L, Sanderson P, Waxenegger H, Mohamed I, Loeb RG (2022). Auditory sequences presented with spearcons support better multiple patient monitoring than single-patient alarms: A preclinical simulation. Human Factors.

[CR35] Gabay Y, Karni A, Banai K (2017) The perceptual learning of time-compressed speech: A comparison of training protocols with different levels of difficulty. *PLoS ONE, 12*(5):e0176488. 10.1371/journal.pone.0176488 10.1371/journal.pone.0176488 PMC5436740 28545039

[CR32] Hughes, R. W. (2014). Auditory distraction: A duplex-mechanism account. *PsyCh Journal*,* 3*(1), 30–41. 10.1002/pchj.44 10.1002/pchj.44 26271638

[CR10] Hughes R, Hurlstone M, Marsh J, Vachon F, Jones D (2013). Cognitive control of auditory distraction: Impact of task difficulty, foreknowledge, and working memory capacity supports duplex-mechanism account. Journal of Experimental Psychology: Human Perception & Performance.

[CR11] Hughes RW, Tremblay S, Jones DM (2005). Disruption by speech of serial short-term memory: The role of changing-state vowels. Psychonomic Bulletin & Review.

[CR12] Jones DM, Macken WJ (1993). Irrelevant tones produce an irrelevant speech effect. Journal of Experimental Psychology: Learning, Memory, and Cognition.

[CR13] Li SYW, Tang T-L, Hickling A, Yau S, Brecknell B, Sanderson PM (2017). Spearcons for patient monitoring: Laboratory investigation comparing earcons and spearcons. Human Factors.

[CR14] Moray N (1959). Attention in dichotic-listening - affective cues and the influence of instructions. Quarterly Journal of Experimental Psychology.

[CR15] Nahum M, Nelken I, Ahissar M (2008). Low-level information and high-level perception: The case of speech in noise. Plos Biology.

[CR16] Naveh-Benjamin M, Kilb A, Maddox GB, Thomas J, Fine HC, Chen TN, Cowan N (2014). Older adults do not notice their names: A new twist to a classic attention task [Article]. Journal of Experimental Psychology-Learning Memory and Cognition.

[CR17] Peirce J, Gray JR, Simpson S, MacAskill M, Höchenberger R, Sogo H, Kastman E, Lindeløv JK (2019). PsychoPy2: Experiments in behavior made easy. Behavior Research Methods.

[CR18] Roche TR, Braun J, Ganter MT, Meybohm P, Herrmann J, Zacharowski K, Raimann FJ, Piekarski F, Spahn DR, Nöthiger CB, Tscholl DW, Said S (2021). Voice alerting as a medical alarm modality for next-generation patient monitoring: A randomised international multicentre trial. British Journal of Anaesthesia.

[CR19] Röer JP, Bell R, Buchner A (2013). Self-relevance increases the irrelevant sound effect: Attentional disruption by one's own name [Article]. Journal of Cognitive Psychology.

[CR20] Röer JP, Bell R, Buchner A (2014). Evidence for habituation of the irrelevant-sound effect on serial recall. Memory & Cognition.

[CR21] Röer JP, Cowan N (2021). A preregistered replication and extension of the cocktail party phenomenon: One's name captures attention, unexpected words do not [Article]. Journal of Experimental Psychology-Learning Memory and Cognition.

[CR22] Ruskin KJ, Hueske-Kraus D (2015). Alarm fatigue: Impacts on patient safety. Current Opinion in Anesthesiology.

[CR23] Sanderson PM, Brecknell B, Leong S, Klueber S, Wolf E, Hickling A, Tang T-L, Bell E, Li SY, Loeb RG (2019). Monitoring vital signs with time-compressed speech. Journal of Experimental Psychology: Applied.

[CR24] Shapiro KL, Caldwell J, Sorensen RE (1997). Personal names and the attentional blink: A visual "cocktail party" effect [Article]. Journal of Experimental Psychology-Human Perception and Performance.

[CR25] Srbinovska M, Salisbury IS, Loeb RG, Sanderson PM (2021). Spearcon compression levels influence the gap in comprehension between untrained and trained listeners. Journal of Experimental Psychology-Applied.

[CR33] Sabic, E., & Chen, J. (2016). *Threshold of spearcon recongition for auditory menus* Proceedings of the 60th Annual Meeting of the Human Factors and Ergonomics Society,

[CR26] Tse C-S, Yap MJ, Chan Y-L, Sze WP, Shaoul C, Lin D (2017). The Chinese lexicon project: A megastudy of lexical decision performance for 25,000+ traditional Chinese two-character compound words. Behavior Research Methods.

[CR34] *The jamovi project*. In. (2022). (Version jamovi 2.3) https://www.jamovi.org

[CR27] Vachon F, Hughes RW, Jones DM (2012). Broken expectations: Violation of expectancies, not novelty, captures auditory attention. Journal of Experimental Psychology: Learning, Memory, and Cognition.

[CR28] Wickens CD (2008). Multiple resources and mental workload. Human Factors.

[CR29] Wood N, Cowan N (1995). The cocktail party phenomenon revisited: How frequent are attention shifts to one's name in an irrelevant auditory channel?. Journal of Experimental Psychology-Learning Memory and Cognition.

[CR30] Woods DD (1995). The alarm problem and directed attention in dynamic fault management. Ergonomics.

[CR31] Yang HS, Wang F, Gu NJ, Gao X, Zhao G (2013). The cognitive advantage for one's own name is not simply familiarity: An eye-tracking study [Article]. Psychonomic Bulletin & Review.

